# Quantitative synchrotron X-ray tomography of the material-tissue interface in rat cortex implanted with neural probes

**DOI:** 10.1038/s41598-019-42544-9

**Published:** 2019-05-21

**Authors:** Thomas Böhm, Kevin Joseph, Matthias Kirsch, Riko Moroni, André Hilger, Markus Osenberg, Ingo Manke, Midori Johnston, Thomas Stieglitz, Ulrich G. Hofmann, Carola A. Haas, Simon Thiele

**Affiliations:** 1 Laboratory for MEMS Applications, IMTEK Department of Microsystems Engineering, University of Freiburg, Georges-Köhler-Allee 103, 79110 Freiburg, Germany; 2 Freiburg Center for Interactive Materials and Bioinspired Technologies (FIT), University of Freiburg, Georges-Köhler-Allee 105, 79110 Freiburg, Germany; 3 BrainLinks-BrainTools, University of Freiburg, Georges-Köhler-Allee 80, 79110 Freiburg, Germany; 4 Neuroelectronic Systems, Dept. of Neurosurgery, Faculty of Medicine, University Medical Center, Engesserstraße 4, 79108 Freiburg, Germany; 5 Department of Neuroanatomy, Institute of Anatomy and Cell Biology, Faculty of Medicine, University of Freiburg, Albertstraße 23, 79104 Freiburg, Germany; 6 Helmholtz Center Berlin for Materials and Energy, Hahn-Meitner-Platz 1, 14109 Berlin, Germany; 7 Institute of Materials Science and Technology, Technical University Berlin, Hardenbergstraße 36, 10623 Berlin, Germany; 8 Experimental Epilepsy Research, Dept. of Neurosurgery, University Medical Center, Breisacher Straße 64, 79106 Freiburg, Germany; 9 Laboratory for Biomedical Microtechnology, IMTEK Department of Microsystems Engineering, University of Freiburg, Georges-Köhler-Allee 102, 79110 Freiburg, Germany; 10 Bernstein Center Freiburg, Hansastraße 9a, 79104 Freiburg, Germany; 11 Forschungszentrum Jülich GmbH, Helmholtz-Institute Erlangen-Nürnberg for Renewable Energy (IEK-11), Egerlandstraße 3, 91058 Erlangen, Germany; 12 Department of Chemical and Biological Engineering, Friedrich-Alexander-Universität Erlangen-Nürnberg, Egerlandstraße 3, 91058 Erlangen, Germany

**Keywords:** Neuroscience, Structural biology

## Abstract

Neural probes provide many options for neuroscientific research and medical purposes. However, these implantable micro devices are not functionally stable over time due to host-probe interactions. Thus, reliable high-resolution characterization methods are required to understand local tissue changes upon implantation. In this work, synchrotron X-ray tomography is employed for the first time to image the interface between brain tissue and an implanted neural probe, showing that this 3D imaging method is capable of resolving probe and surrounding tissue at a resolution of about 1 micrometer. Unstained tissue provides sufficient contrast to identify electrode sites on the probe, cells, and blood vessels within tomograms. Exemplarily, we show that it is possible to quantify characteristics of the interaction region between probe and tissue, like the blood supply system. Our first-time study demonstrates a way for simultaneous 3D investigation of brain tissue with implanted probe, providing information beyond what was hitherto possible.

## Introduction

Advancements in neuroscience rely on gathering morphological and functional information on the properties of brain tissue. To comprehensively elucidate morphological and electrophysiological characteristics, an investigation of brain tissue requires imaging methods capable of analyzing tissue in three dimensions, and on different length scales, as well as measuring neuronal activity^1^. While imaging at magnifications stretching over several orders of magnitude and acquisition of electrophysiological data can be performed independently using a variety of established techniques, the most challenging approach is to correlate single unit activity to microscale morphology acquired at the very same location. A common, very accurate way of characterizing neuronal activity up to single unit activity is achieved by implanting neural probes into the brain, mostly equipped with a multitude of microelectrodes^2,3^. However, the foreign body response upon implantation leads to a spatially confined inflammatory response and glial scarring, potentially leading to probe failure^4^. It is therefore desirable to optimize probe designs towards reducing the foreign body response in order to maintain high quality signals over long periods of time^5,6^. Thus, an imaging approach providing a 3D reconstruction of the interface between brain and implanted probe is sought for studying histology and for evaluating local changes in morphology upon implantation to improve probe design and implantation techniques in the long run.

Imaging of tissue features in the size range of tens of microns is easily accomplished with standard light microscopy, yet techniques with a higher resolution are required to collect information below the micrometer-scale. Confocal laser scanning microscopy is one of the methods capable of 3D imaging at a spatial resolution of less than one micrometer^7^. Recent advances in this field even allow optical microscopes to circumvent the classical resolution limit and reach the two-digit nanometer range^8^. However, tissue preparation, staining, and imaging itself remain time-consuming. Imaging of tissue volumes larger than the typical capabilities of confocal microscopes (up to 100 µm in z-direction^9^) can only be performed with specialized techniques such as multiphoton microscopy^10^, yielding imaging depths of up to 1 mm in brain tissue^11^, or with tissue clearing methods such as CLARITY that enable researchers to analyze large tissue blocks or even entire organs^12^.

Imaging of tissue at resolution levels far beyond the limits of optical microscopy is possible with electron microscopy^13^. Especially scanning electron microscopy (SEM) has proven itself to be an extremely valuable tool for researchers^14^. While not being able to deliver the outstanding sub-nanometer spatial resolution of transmission electron microscopy^15^, SEM provides the possibility to image the surface of embedded tissue blocks. Serial block face SEM and focused ion beam SEM achieve a spatial resolution of single-digit nanometer pixel sizes and can be applied to more than a single section^13,16–19^. Therefore, they also meet the requirements for volume rendering of cerebral microstructures. Recent methods and devices like array tomography and the automated tape collecting ultramicrotome (ATUMtome) provide an alternative solution for 3D imaging at SEM resolution levels. Array tomography uses serial sections of resin embedded tissue which are stained sequentially for confocal microscopy and subsequently contrasted for SEM^20^. Yet classical array tomography requires manual collection of ribbons of ultra-thin sections, which is limited in the number of sections obtainable and is prone to induce errors^21^. The ATUMtome, in contrast, automates sectioning and sample collection, allowing the collection of more than one thousand sequential ultra-thin sections without interruption^14,22^. In concert with multi-beam SEM devices, the imaging process is thus accelerated and automated^23^. Despite these advances imaging is still time consuming and the sampling volume is very limited especially when imaging at a high spatial resolution is required.

Further challenges for light and electron microscopy are posed by tissue analysis with an implanted neural probe, although tissue preparation and imaging methods are well established and highly standardized^24,25^. In order to use standard embedding and staining methods, most probes need to be removed prior to tissue processing, making it difficult to precisely reconstruct their original position in retrospect. Although probe removal overall is advantageous for tissue preparation, it might induce further damage to the sample as cells adhering to the interface are lost and the extraction forces further distort the remaining tissue. Flexible probes may remain within the tissue during preparation, but on the other hand they often detach from the tissue upon fixation or sectioning. Furthermore, artifacts may result from sectioning of materials with different mechanical properties. A possible way to circumvent these issues is to perform tomography without physical sectioning, which is possible using e.g. magnetic resonance imaging (MRI), or computed tomography (CT). MRI is a fast developing tomography approach that can be performed *in vivo* and without detrimental ionizing radiation. While MRI can deliver large fields of view at magnetic field strengths of 1.5 T and 3.0 T in clinical practice, the spatial resolution is limited. Even field strengths as large as 10 T do not allow a spatial resolution below several microns but limit the size of the investigated object due to the bore diameter^26^. Further, MRI scans suffer from massive imaging artifacts occurring in the presence of metals and other materials with susceptibilities that do not match the ones of brain tissue^27^. More seriously, potential hazards due to implant movement by magnetic forces or heating of implants might occur^28^. Although the spatial resolution is expected to increase within the next years, the high magnetic field strengths required for high resolution MRI also increase the effect of metallic artifacts on data quality^29,30^.

A rather new development is the use of high resolution synchrotron X-ray tomography (µCT) to investigate biological samples and in particular brain tissue at and even slightly below the micrometer scale^31^. By employing a monochromatic X-ray beam from a synchrotron instead of lab-scale X-ray tomographs, the 3D reconstruction of soft tissues with phase contrast imaging is feasible without the use of any contrast agents. Although µCT cannot provide the specificity of immunohistochemistry (IHC) towards specific cell types and proteins by the variety of available IHC-stainings, it offers a remarkable morphological resolution. Hieber *et al*. showed an impressive example of the capabilities of this technique by imaging Purkinje cells within a fixed and embedded, but unstained human cerebellum^32^. X-ray tomography can be adapted towards the resolution and field of view that is needed for the desired application. Therefore, so-called nano-CT with a resolution of about 50 nm may be performed^33^. However, nano-CT yields its high resolution at the cost of a substantially smaller field of view, typically only covering a few microns. Tomograms of more than 1 mm³ can be easily acquired with µCT within minutes instead of days or weeks that need to be invested when performing physical sectioning methods such as SEM-imaging of serial sections or micro-optical sectioning tomography^14,34^. Compared to 3D imaging methods such as confocal or multiphoton microscopy, which are limited by their penetration depth, available staining techniques, or might require transgenic animals for imaging tissue features, µCT can complement tissue analyses with these unique characteristics. In addition, µCT allows correlative imaging by combining X-ray datasets e.g. with electron micrographs after the non-destructive synchrotron X-ray tomography^35,36^. Thus, µCT is a valuable tool for neuroscientists and has been adopted quickly by the community within the last few years^32,35,37–39^.

Despite these advantageous characteristics of synchrotron X-ray tomography, it is still demanding to analyze materials with contradictory features at the same time. It is known that samples containing phases with different attenuation coefficients, like metals and carbon based materials, are difficult to be imaged simultaneously: e.g. when imaging battery electrodes that contain cobalt-oxide and carbon, the X-ray energy has to be chosen high enough to image through the cobalt, while the carbon phase at these settings becomes effectively invisible^40,41^. Thus, for X-ray tomography, samples with different, but similar absorption contrast of all components are advantageous in order to visualize not only a high-contrast phase but the complete structure of the specimen. In the case of imaging the interface between brain and an implanted neural probe, the tissue is carbon-rich and therefore shows relatively weak X-ray absorption. In contrast, metal components of a neural probe represent a highly absorbing phase that potentially interferes and masks the structure of the surrounding biological tissue, although metallic electrode sites and interconnection tracks are relatively delicate compared to the organic and inorganic substrate and insulation materials of these probes^42–44^. Thus, it was not clear whether this combination of materials could be imaged by µCT at a sufficient signal to noise ratio (SNR) to provide useful information on the tissue interface. However, in this work we show that µCT is indeed capable of imaging the interface between brain and probe, thereby complementing more classical techniques for the analysis of the material-tissue interface.

## Results and Discussion

### Tomographic imaging

**Figure 1:**
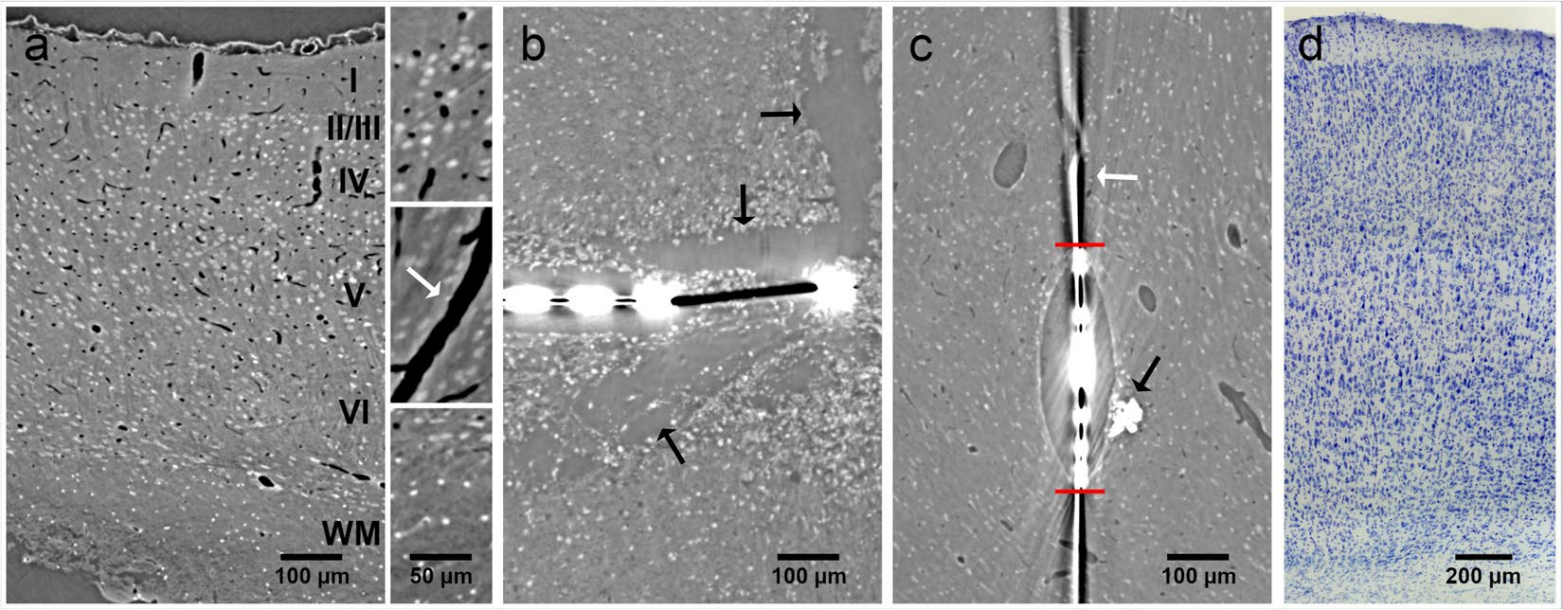
Virtual sections of X-ray tomograms. (**a**) Control sample: virtual vertical section of rat cortex tissue without probe. The small images on the right are 2x magnified views of this tomogram. Blood vessels are clearly visible (dark profiles, white arrow in the right center image) and cortex layering can be visualized by different size, shape, and density of cells. The upper image on the right shows layers II/III. The lower image shows the transition from layer VI to white matter. (**b**) Virtual vertical section of rat cortex tissue 4 hpi. The image is rotated 90° counterclockwise (pial surface on the left). Saturated pixels represent the electrodes of the probe. Black interconnections between the electrodes occur due to the missing wedge artifact. Massive tissue damage and ruptures surround the probe, indicated by black arrows. (**c**) Virtual transversal section of rat cortex tissue 12 wpi. Black and white ray-like structures from the brain-probe are due to the missing wedge artifact (white arrow). Transition from probe to tissue that is covered by the missing wedge artifact is marked with red horizontal lines. Note the elliptic cavity surrounding the probe. The black arrow highlights the calcification visible at the right outer rim of the cavity. (**d**) Light microscopic image of a vertical section of rat cortex tissue after Nissl-staining.

**Figure 2:**
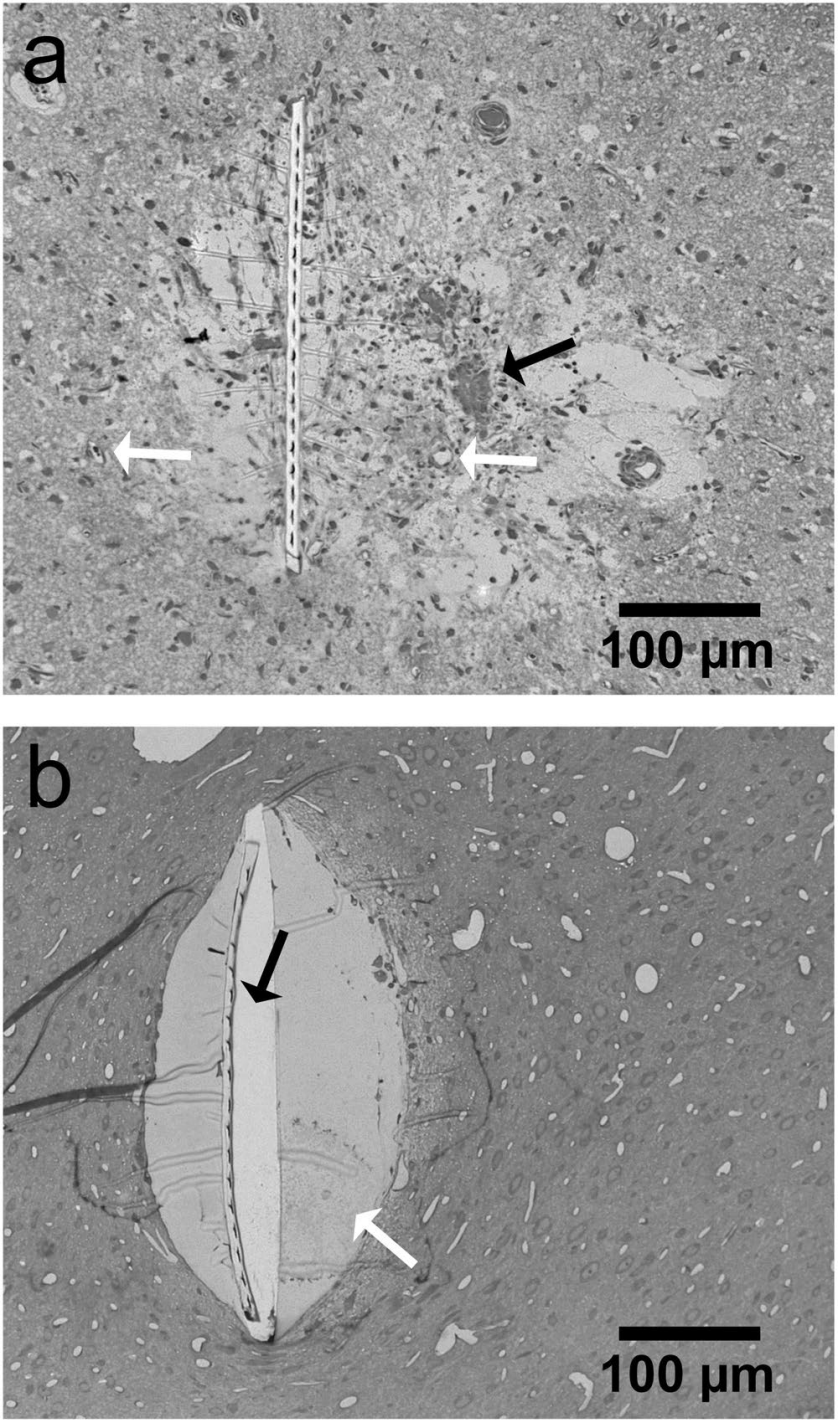
Semi-thin transversal sections of samples after X-ray tomography. Light microscopic images after staining with toluidine blue are shown. (**a**) Rat cortex tissue 4 hpi. The black arrow indicates signs of hemorrhage. White arrows show blood vessels. (**b**) Rat cortex tissue 12 wpi. The white arrow indicates epoxy resin (see results section for further discussion) and the black arrow indicates detachment of the neural probe from the resin embedded sample.

Virtual sections of rat cortex tissue were obtained by µCT at a resolution level comparable to light microscopy (Fig. 1a,d). Control tissue without an implanted probe allowed a distinction between cells and blood vessels, although no contrast agent had been used. Note that the original orientation of sagittal and coronal plane was not preserved in the tomograms. Thus, virtual sections orthogonal to the transversal plane are described as vertical sections in the following. Within a virtual vertical section of control tissue, even the cortical layering could be identified (Fig. 1a). As expected, the image quality decreased in the presence of a high-contrast material within the sample (acute and chronic implantation of a flexible neural probe, Fig. 1b,c). Nevertheless, not only structures of the probe but also of the tissue could be visualized. Massive tissue damage was visible in an acute implantation sample by large ruptures surrounding the probe (4 h post implantation (hpi), Fig. 1b). Since the implantation was performed with a rigid shuttle-device in order to insert the flexible polyimide-based probe into the tissue, we expected tissue damage in the immediate vicinity of the probe. Although it was not possible to differentiate between damaged nervous tissue and bleeding from damaged blood vessels, disruption of the normal cytoarchitecture could be clearly identified (Fig. 1b). A semi-thin toluidine blue stained section of the 4 hpi sample (Fig. 2a) supported this finding by showing features of tissue damage and likely residues of blood, which indicate hemorrhage (black arrow in Fig. 2a). Although blood vessels could still be identified in the semi-thin section (white arrows in Fig. 2a), they largely lost their lumen, which explains why they could not be identified in the X-ray tomography. Local breach of the blood-brain-barrier promotes a pro-inflammatory environment and has been correlated with increased neural probe failure rates^45^. With the ability to investigate the implantation site in 3D acutely after probe insertion, µCT is well suited to compare different implantation techniques with respect to affected volume and severity of tissue damage by probe insertion. This is of interest especially when comparing standard implantation techniques of flexible microelectrodes using stiff rods or stiffening agents as guides for the probe with new developments such as fluidic microdrives that allow implantation of flexible probes by tension force without an increase in stiffness or size of the probe^46^.

X-ray imaging of a 12 weeks post implantation (wpi) chronic sample (Fig. 1c) provided histologic information almost at the quality level of the control sample without implanted probe (Fig. 1a). However, artifacts that originated from high-contrast metal components of the probe were visible in one direction (white arrow in Fig. 1c): highly similar to the so-called missing wedge artifact in electron tomography^47^, which describes the lack of imaging data at certain parts of the imaged volume, there was a lack of information in the X-ray tomogram along this direction. In electron tomography, the missing wedge artifact originates from a limited sample tilt angle, whereas in the X-ray tomogram the missing information was not due to insufficient sample rotation, but due to the metal components within the sample. We attribute this artifact to beam absorption by the metal parts of the probes as it only occurred at rotational angles with a serial accumulation of metal. Consequently, the signal to noise ratio along relatively large amounts of metal was reduced. Nevertheless, the image quality was almost unimpaired perpendicular to this direction on both sides of the probe and therefore provided valuable information about the interface between brain tissue and neural probe. A higher cell density as compared to the rest of the parenchyma was partially visible surrounding the elliptically shaped cell-free volume around the probe (Supplementary Fig. 1). We attribute this finding to the outcome of a sterile inflammatory response at the implantation site and the local encapsulation of a foreign body referred to as a glial scar^4^. Since we were unable to identify unambiguously from the X-ray tomography data whether the cell-free ellipsoidal area around the probe (Fig. 1c) was infiltrated by epoxy resin or not, it potentially resulted from tissue shrinkage during fixation or from shrinkage of the epoxy resin during polymerization. By embedding a probe in epoxy resin and imaging a cross-section by light microscopy we could exclude the latter as in this case no artifact like the one seen in the µCT tomogram appeared (Supplementary Fig. 2). In addition, a semi-thin section of the 12 wpi sample clearly identified this cell-free area as epoxy resin (white arrow in Fig. 2b). Therefore, the most plausible explanation for this elliptic cavity is tissue shrinkage during fixation and dehydration that caused the tissue to become locally detached from the probe, leaving open a hole that was later filled with epoxy resin in the embedding step.

**Figure 3:**
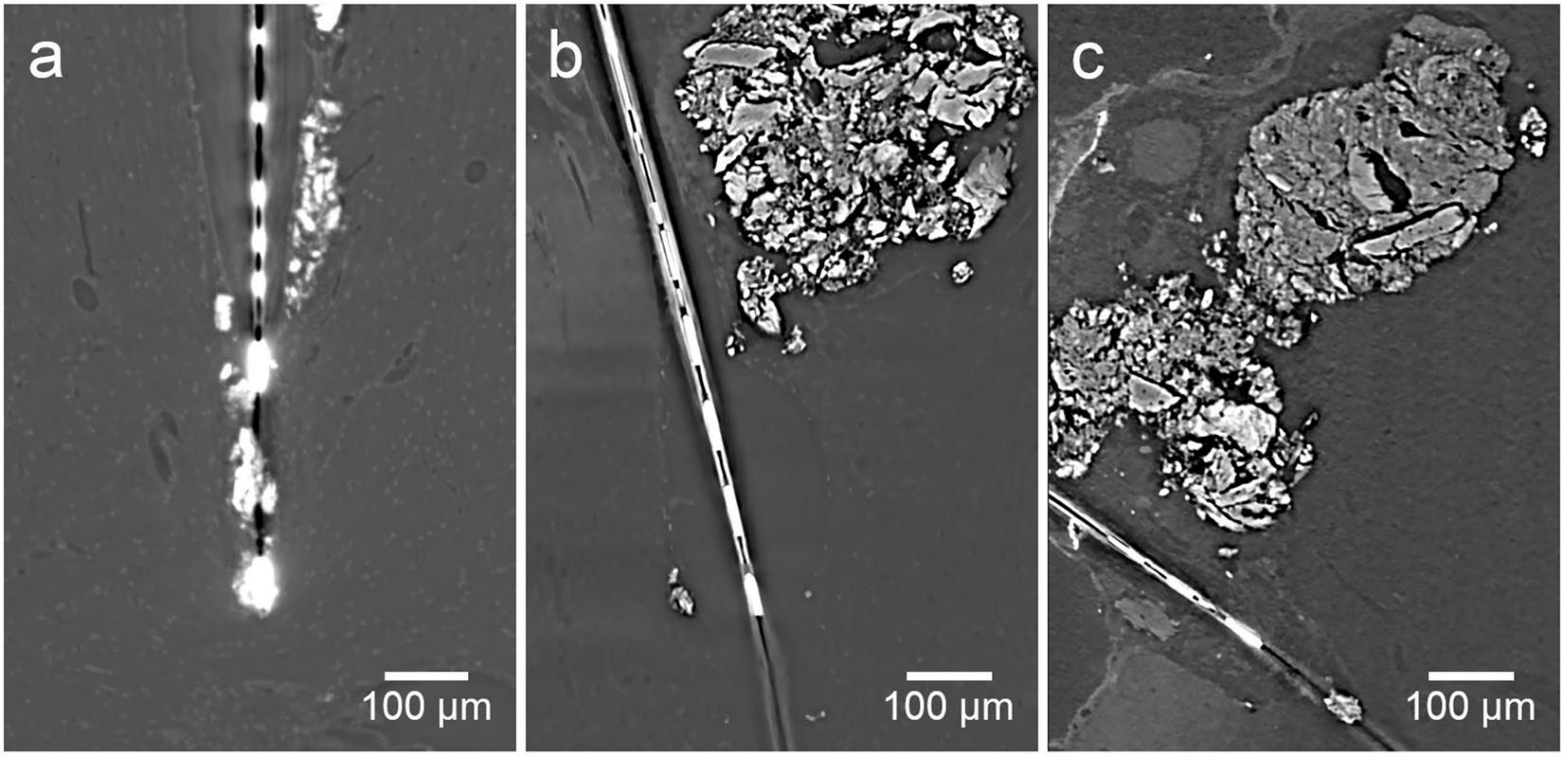
Virtual sections of X-ray tomograms with calcifications in close vicinity to implanted neural probes. Depicted are the tissue-probe interfaces of the 12 wpi sample (**a**), 2 wpi sample a (**b**), and 2 wpi sample b (**c**). Bright areas surrounding the probe represent calcifications that provide a significantly higher X-ray contrast than brain tissue.

**Figure 4:**
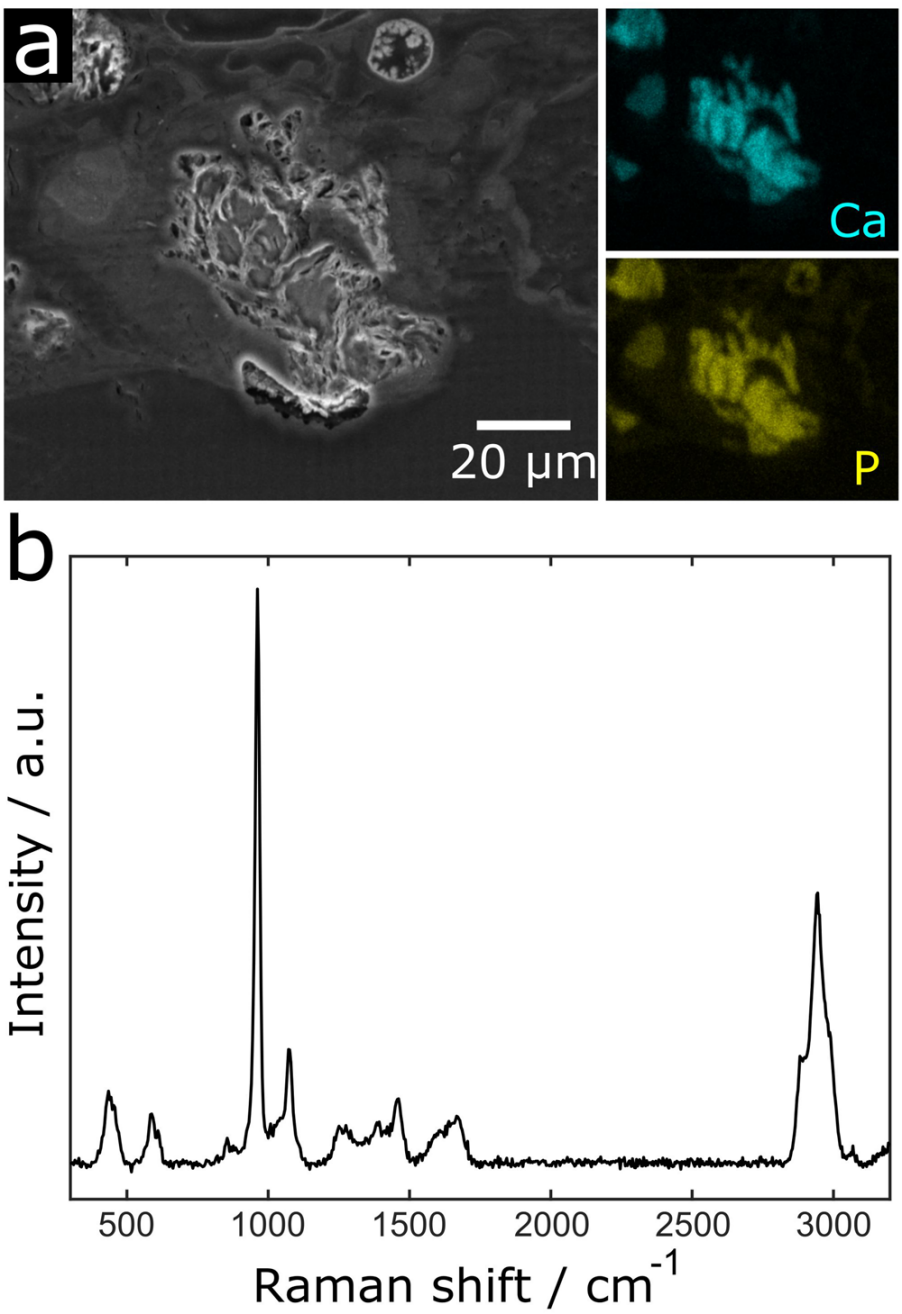
Raman and Energy dispersive X-ray spectroscopy of 2 wpi sample a at the calcification. (**a**) SEM-image of the sample surface (left) with EDX mappings of the same region for Ca (right upper image, cyan) and P (right lower image, yellow). The latter clearly identify the structure as accumulations of Ca und P. (**b**) The Raman spectrum of the calcification shows the peaks typical for hydroxyapatite at around 435, 590, 960, and 1075 cm^−1^ ^69^. Furthermore, protein-related peaks are recognizable by the CH-stretching region between 2800 and 3050 cm^−1^ as well as by the amide-peak with its maximum at around 1670 cm^−1^ ^70,71^.

A further interesting finding was the occurrence of structures at the brain probe interface that exhibited a remarkably higher X-ray contrast than brain tissue (black arrow in Fig. 1c). These structures were visible in the chronic implantation sample (Fig. 3a) as well as in two additional tissue samples at 2 wpi (Fig. 3b,c). Due to the high X-ray contrast of these structures we hypothesized that these structures consisted of calcium. It was shown before that calcifications can occur in the brain in pathologic conditions such as epilepsy and trauma^48^. While diseases like neoplasms or vascular lesions can lead to so-called brain stones in the size of centimeters^49^, small calcifications are even visible under physiologic conditions^50^. Hemorrhage, leakage of the blood-brain-barrier, and chronic inflammation upon implantation were therefore potential causes for local calcifications. To further analyze these structures we prepared a cross-section of one of the samples with such a structure (2 wpi, sample a) and using energy dispersive X-ray spectroscopy (EDX) we could confirm that there were local accumulations of calcium and phosphor at these sites (Fig. 4a). This result was further strengthened by Raman spectroscopy, which identified these structures as hydroxyapatite (Fig. 4b). Hydroxyapatite is clearly visible in X-ray tomography but may be missed by tissue analyses that use IHC. Nevertheless, a report from 1995 described two cases of patients suffering from epilepsy who were implanted with depth electrodes. Calcification at the implantation sites occurred and could be verified even two years post explantation^51^. Interestingly, at the cross-section prepared for Raman- and EDX-analyses, which was generated at the upmost sample position that showed the calcification, histologic investigation revealed that the calcification protruded from brain tissue. Potential explanations for this finding are shrinkage of soft tissue upon processing (compare Fig. 2b) or bone fragments as remnants from the implantation procedure.

### Segmentation of the samples

**Figure 5:**
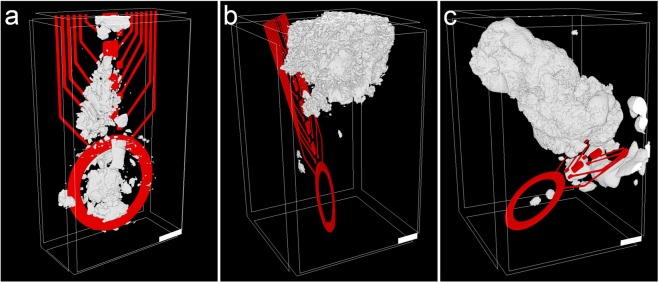
Segmentation and 3D reconstruction of implanted neural probes with adjacent calcifications. Metal structures of the probes (red) were easily recognizable by µCT. Also, the calcifications (white) provided a higher X-ray contrast than the surrounding tissue, allowing successful segmentation even in close proximity to the imaging artifacts caused by the probe. (**a**) tomogram of tissue 12 wpi. (**b**,**c**) are tomograms of tissue 2 wpi (samples a and b). Scale bars in all images equal 100 µm.

In addition to the qualitative information from virtual sections of X-ray tomograms, quantitative data was obtained after segmenting the 3D datasets. In order to reliably assign voxels within a dataset to a certain structure, the contrast within the data needs to be sufficient. Electrode tracks of the brain-probes and calcifications within the tomograms could be easily identified due to their high X-ray contrast. Thus, the segmentation of probe and calcification could be performed by a simple manual grayscale-thresholding (Fig. 5). However, the probe substrate remained invisible because of the strong signals of the metal lines on the probes.

**Figure 6:**
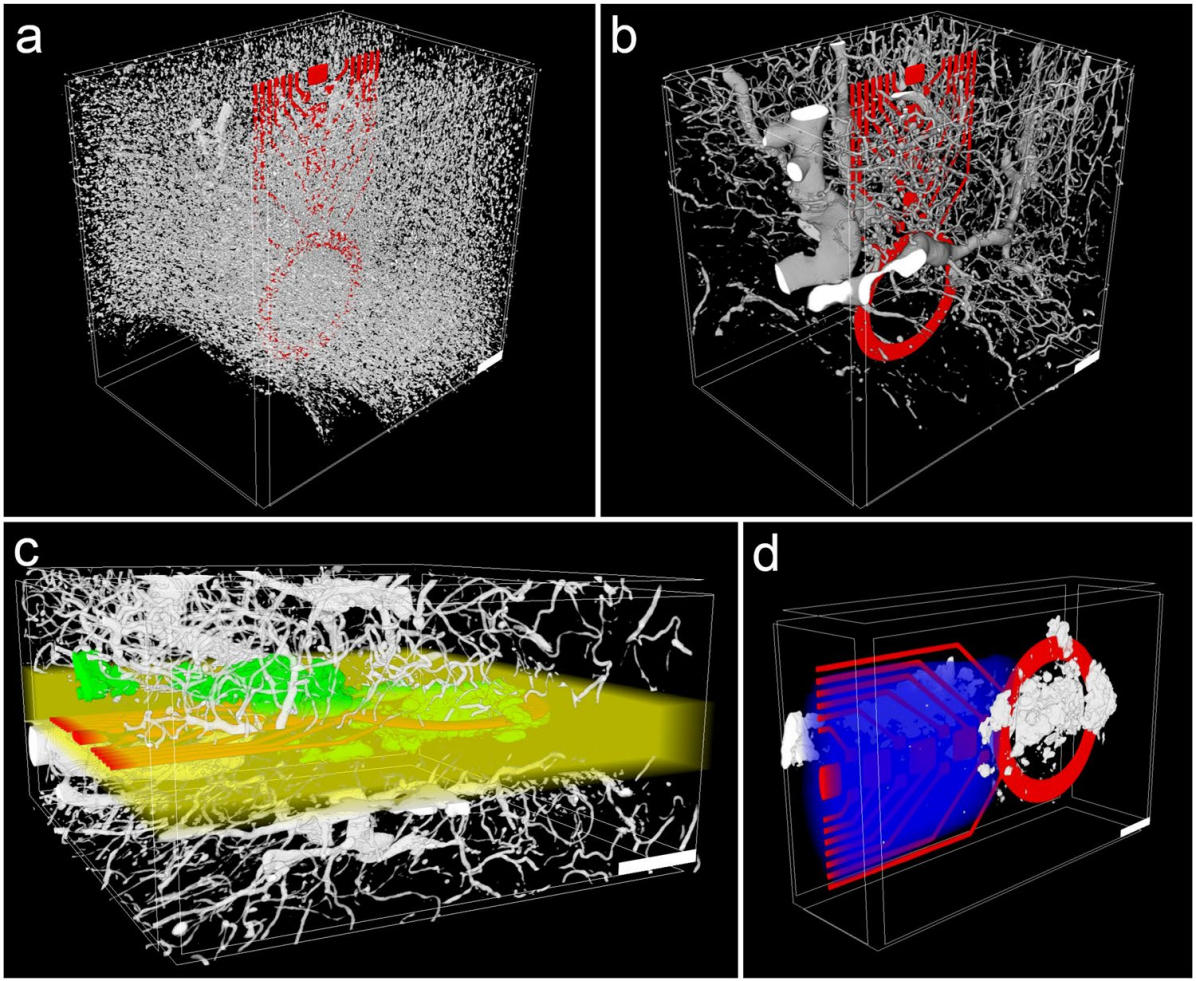
Segmentation and 3D reconstruction of cells, blood vessels, calcification, and cavity in the surrounding of a neural probe in rat cortex tissue 12 wpi. (**a**) Cells (white) in the vicinity of the neural probe (red). (**b**) Blood vessels in the volume shown in (**a**). (**c**) Blood vessels (white) and calcification (green). The yellow volume was excluded from the segmentation of cells and vasculature because of the missing wedge artifact. (**d**) Segmentation of the cavity (blue) that occurs along the probe shaft. Scale bars in all images equal 100 µm.

**Figure 7:**
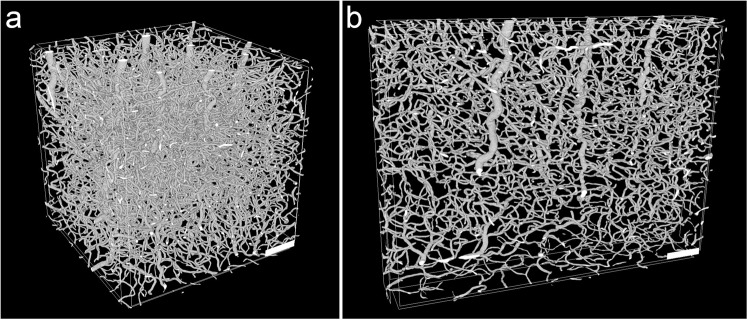
Segmentation and 3D reconstruction of cerebral vasculature in tissue without probe. (**a**) Blood vessel structure in rat cortex imaged by µCT. The displayed data is a part of the tomogram and encompasses a volume of 500 × 500 × 500 µm³. (**b**) Vertical section of the same tomogram. The section volume is 800 × 600 × 100 µm³. Scale bars in both images equal 100 µm.

Dyer *et al*. demonstrated an automated segmentation of cells and blood vessels with µCT^35^, which in general is a good indicator for a large contrast difference between the single structures. However, with decreasing SNR and more artifacts within the data, it is difficult to find a suitable automated approach for a segmentation of 3D datasets at a reasonable quality. A lower SNR occurred in samples containing metal that caused missing wedge artifacts, leading to an inconsistent grayscale baseline in tomograms (Fig. 1c). Thus, for the 12 wpi chronic sample we also performed a manual segmentation after contrast enhancement of cells and blood vessels (Fig. 6). Note that due to imaging artifacts introduced by metal tracks of the probe, image segmentation and subsequent analysis are hindered in the direct surrounding of a neural probe. For example, in the upper left part of Fig. 6a, a segmentation error occurred in form of a small part of a blood vessel close to the probe that was falsely assigned as cells. Cell- and blood vessel-free areas in the lower left parts of Fig. 6a,b are not caused by imaging artifacts, but represent the end of the sample. While the SNR of the 2 wpi samples was sufficient for the identification of probe and calcification, the vasculature and cells were not adequately represented in the tomograms (Supplementary Fig. 3). For the control sample without staining we were able to confirm the results of Dyer *et al*.^35^; a segmentation of the blood vessels was easily performed within this sample due to a high SNR and a lack of imaging artifacts (Fig. 7). Using multiphoton microscopy, it has been shown that it is possible to investigate the local vasculature in the surrounding of a neural probe *in vivo*^52,53^. Compared to our µCT approach, the light microscopic technique harbors the advantage of performing an *in vivo* analysis without artifacts from tissue processing. On the other hand, multiphoton microscopy requires sophisticated experimental setups like a cranial glass window for imaging and it is limited in imaging depth. Although µCT in the setup used in this work is not capable of replacing standard histological analyses due to the limited access to synchrotron facilities, it offers an interesting and valuable complement to other imaging methods.

From segmented datasets a quantitative analysis regarding e.g. the volume and distribution of a specific structure could be performed. However, it has to be taken into account that results from a quantitative evaluation of processed tissue do not represent the *in vivo* situation. It has been shown that tissue fixation with paraformaldehyde followed by paraffin embedding leads to tissue shrinkage of more than 50%^54^. The effect of shrinkage has very likely already revealed itself in the cavity surrounding the neural probe in the 12 wpi sample (Figs 1c and 2b). Without 3D data and fiducial markers from pre and post tissue processing it is not possible to determine one specific factor by which the complete dataset needs to be expanded to try to restore the original volume. Thus, any quantitative results from 2D or 3D imaging of tissue after fixation and embedding have to be interpreted in the light of this processing artifact. Another option to minimize the effect of tissue shrinkage and deformation upon tissue processing is using a fixative that leads to less tissue alterations in the first place. For example, Wehrl *et al*. found that using a zinc-based fixative limits tissue shrinkage to 33.5%, which is substantially smaller than the volume loss upon tissue fixation with paraformaldehyde^54^.

### Quantification of the results

A rather straightforward quantification of tomographic data is the analysis of distances between different detected structures as well as their respective volumes or volume fractions. The latter two can be calculated easily as long as the voxel dimensions are known. However, due to tissue shrinkage, the values obtained are not readily comparable with results obtained from tissue that was processed differently. In the 12 wpi sample, a proportion of the volume directly adjacent to the probe had to be excluded from the segmentation of cells and blood vessels (Fig. 6c) because of the missing wedge artifact and therefore was also removed from the volume used for tissue parameter quantifications. This volume encompassed 5.85∙10^7^ µm³ (14% of the investigated tissue volume), with a distance of less than 46 µm to the sides of the probe shaft. Note that the size of this artifact depends on probe type and µCT data quality. The necessity to remove parts from analysis depends on the structures to be investigated. For example, due to a very high signal to noise ratio, calcifications and metal tracks of neural probes could be segmented and quantified without any restrictions due to imaging artifacts. Further, the volume that has to be excluded from quantification varies with the segmentation approach, with manual segmentation providing the option to reduce the size of this volume, but increasing the time required for segmentation.

First, we compared the vasculature between control tissue and the 12 wpi tomogram. For data evaluation, it has to be taken into account that the vasculature within the brain is not homogeneously distributed, but differs remarkably between brain regions. Especially the transition from gray to white matter shows a drastic reduction in blood vessel density^55^. Therefore, the exact anatomical localization of a sample is crucial when comparing the vasculature between different samples. In our case the control tissue volume spanned the six cortical layers and also included a part of the white matter (Fig. 1a). The 12 wpi sample was imaged with the tip of the cortical probe still being part of the tomogram, which resulted in a field of view that did not include the pial surface. Within this tomogram the cortical layering was not as clearly recognizable as in the control tissue. Thus, the volume contributions of cortex and white matter could not be determined unambiguously for this tomogram. Under physiological conditions, a lower vascular density can be expected in case the contribution of white matter is larger than that of cortex as compared to the tomogram of tissue without probe. We found that the volume proportion occupied by blood vessels was slightly reduced in the chronic implantation sample (2.0%) as compared to the control tissue (2.3%), but still within the range of previously published studies in rat (2–2.5%^56^) and mouse cortex (2.6%^34^).

**Figure 8:**
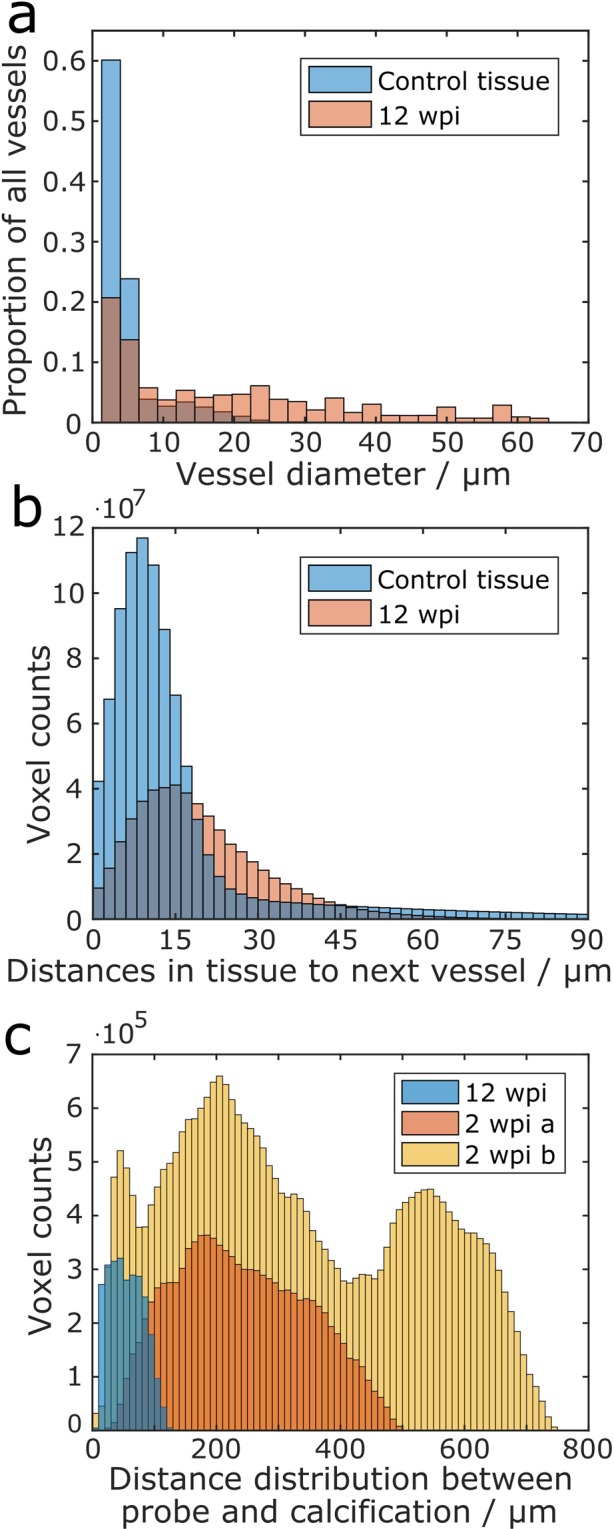
Quantitative analysis of calcifications and blood vessels within analyzed tissue volumes. (**a**) Size distribution of vessels in control tissue and in tissue 12 wpi. Provided are histograms that display the diameters of blood vessels in the investigated tissue volumes. (**b**) Histograms of distances of all voxels to the closest vessel in control tissue and tissue 12 wpi. (**c**) Distance distribution between calcification and probe in three tomograms with calcifications. The distances between all voxels that were segmented as part of calcifications to the closest voxel of the neural probe are shown. For all histograms in this figure, n = 1.

Cavaglia *et al*. found that the density of capillaries is up to fivefold higher in the neocortex than in the white matter of rats^55^. Thus, not only the overall volume proportion, but also the size distribution of the blood vessels is a relevant parameter for the analysis of cerebral vasculature. We used a custom-built MATLAB code performing a pore size distribution analysis within the 3D datasets to determine the diameter distribution of the vessels^57^. Virtual spheres were projected into all volumes identified as vessels with the maximum diameter that could be achieved without protruding into the surrounding space at any point. A simplified visualization of this computation is presented in Supplementary Fig. 4. The size distribution of vessels is provided in Fig. 8a. An apparent shift towards larger diameters was found in the 12 wpi chronic implant sample that led to an almost equal overall vessel volume proportion in comparison to the control tissue, yet with fewer microvessels and more vessels larger than 10 µm in diameter. This finding suggests that there is a considerable vascular reorganization following probe implantation. However, a larger study with more samples and tomograms at identical cortical positions is required in order to validate this hypothesis.

Another interesting parameter for the analysis of changes in the vasculature is the distance between single blood vessels, since this parameter determines the diffusion radius of nutrients within a tissue. We evaluated the distance between vessels in the control tissue as well as in the 12 wpi sample (Fig. 8b) using a distance transform of the respective vessel segmentation. An illustration of the principle is provided in Supplementary Fig. 5. We found that for cortex tissue without probe, the median distance of every voxel in the tissue towards the next vessel was 10.7 µm. This result is in agreement with data obtained by Wu *et al*.^34^, who determined the same parameter in mouse cortex tissue (12 to 17 µm), however after applying a correction to compensate for tissue shrinkage. For the 12 wpi sample the same parameter was shifted towards higher values with a median distance of 17.2 µm to the next vessel. Considering that the volume occupied by blood vessels in the chronic implantation sample was only slightly reduced (11% less than in the control tissue) while the median distance to the next vessel was increased by 61% (Fig. 8b), a shift from smaller towards larger blood vessels in the 12 wpi sample can also be concluded from this data.

Regarding the calcifications in the 12 wpi sample, we found that these structures occupied a volume of 1.78∙10^6^ µm³. Calcifications also occurred in two 2 wpi samples (Figs 3, 5). Within these samples this structure was an order of magnitude larger than in the long-term sample, consuming 1.56∙10^7^ µm³ in 2 wpi sample a and 4.39∙10^7^ µm³ in 2 wpi sample b. Also, the spatial distribution of these structures could be determined. A distance transform of the probe segmentation allowed evaluating the distance of every single calcification voxel to the probe (Fig. 8c). Quantification of the calcification volumes and distance calculations could be performed without interference from the missing wedge artifact due to the high contrast of these structures. The calcification was very close to the probe in the 12 wpi sample, with a median distance of 53.7 µm. For the 2 wpi samples, the median distances between the calcification and the probe were considerably higher with 232.3 µm (2 wpi a) and 296.3 µm (2 wpi b).

## Conclusion

In several studies µCT has been employed as a powerful tool for the volume analysis of brain tissue down to sub-micron resolution^32,35,39^. We show for the first time that this imaging technique can also successfully display brain tissue in the presence of an implanted neural probe. Although metal components of the probes led to imaging artifacts, the data quality was sufficient for a distinction between cells and blood vessels in the direct vicinity of the probe. Thus, the proof was made that µCT offers the power to investigate local tissue alterations upon probe implantation without the need for staining or physical sectioning. By imaging the material-tissue interface of a neural probe, synchrotron X-ray tomography enables an analysis of tissue in 3D acutely post implantation or after longer time periods, providing the opportunity to compare how different implantation techniques and implant types influence their immediate surroundings. The metal layers of the probes used in this work are relatively thick (300 nm) in comparison to e.g. only 100 nm thin metal layers which have been successfully used in other probe designs^52^. Thinner metallization will reduce the artifacts seen in X-ray tomography and is therefore supposed to improve data quality of tissue imaged directly at the interface. This holds true even when several connection lines are stacked, e.g. as in the probes shown by Luan *et al*.^52^, since the peak metal volume that needs to be penetrated by the X-ray beam will be reduced. Consequently, the missing wedge artifact is expected to be less pronounced, in comparison to when connection lines are placed in the same plane as it is the case in this work. Our non-destructive 3D imaging method also revealed an artifact that appeared most likely due to tissue shrinkage, namely an elliptically shaped cavity surrounding the probe. Unfortunately, tissue shrinkage due to fixation and embedding is inevitable^54^, and potentially separates the interface between probe and tissue. At the current state of the art, *in vivo* µCT with resolution and signal quality as shown in this work is still out of reach. Nevertheless, Töpperwien *et al*. have recently shown that it is possible to obtain tomographic data from stained, yet still hydrated brain slices by synchrotron X-ray tomography^58^. Thus, an important future question will be whether fixation and embedding can be circumvented to make a direct investigation of the brain-probe interface in hydrated tissue possible.

While µCT is capable of identifying single cells, the spatial resolution is not sufficient to further distinguish between different cell types. The latter would require a resolution in the nanometer scale or the use of specific stainings^35^. We thus focused on quantitative analyses of the vasculature within the investigated samples. While in acutely implanted tissue the circulatory system showed signs of disruption, clearly recognizable blood vessels were found in control tissue as well as in chronically implanted tissue. The volume occupied by the vasculature was similar in both samples and in accordance with already published data (approx. 2–2.5%^34,56^). However, the chronic implantation sample differed from control tissue with respect to the size distribution of the vessels, as well as the distance between vessels, showing more vessels with a diameter of more than 10 µm and fewer microvessels, and accordingly a higher median distance between vessels, which we were able to quantify from the µCT datasets. The field of view of the chronic implantation sample covered both cortex and white matter, whereas the tomogram of the control sample spanned a small contribution of white matter and the complete cortex, which is known to have a higher density of capillaries than white matter^55^. While the different regions of imaging render an unambiguous interpretation of this outcome difficult, our data still suggests that angiogenesis occurred at the implant site. A study with a higher number of samples and imaged within directly comparable regions is required in order to fully support this finding. Nevertheless, our proof of principle study can be the basis of future investigations in this field. Furthermore, µCT revealed the presence of calcifications close to the implantation sites. Due to the relatively high X-ray contrast of hydroxyapatite these structures were clearly visible within X-ray tomograms, yet they may remain unrecognized by standard procedures for tissue analysis. Thus, local calcifications in brain tissue around implanted probes may be a more frequent phenomenon than the rare reports in literature imply.

A comprehensive understanding of processes at the material-tissue interface of neural interfaces is desirable and mandatory to transfer results from neuroscientific research into clinical applications from which patients can benefit for their life-time. While synchrotron X-ray imaging is not suited as a routine analysis method since access to beamtime is limited, synchrotron µCT still harbors the potential to complement established methods and to add correlative information from *in vivo* multiphoton microscopy before µCT or IHC and SEM after µCT into the big picture of information about the local tissue reactions after neural probe implantation. Only with a more detailed and complete picture of the foreign body reaction with respect to its temporal and spatial components, knowledge can be generated and translated into robust and reliable neural implants for clinical applications.

## Material and Methods

### Probes

Flexible neural probes were based on a thin polyimide substrate with platinum thin-film metallization sandwiched between the polyimide layers, fabricated as described previously^5,59^. In brief, a 6 µm polyimide layer was deposited on a silicon wafer. To promote adhesion of the metal interconnects, 50 nm of silicon carbide were deposited on the polyimide layer by chemical vapor deposition^60,61^. Subsequently, 300 nm of platinum were sputtered onto the silicon carbide, followed by another 30 nm of silicon carbide and 10 nm of diamond-like carbon. For manufacturing the electrode contacts, 100 nm of iridium and subsequently 800 nm of iridium oxide were selectively deposited onto the substrate and photo-lithographically patterned. A second layer of polyimide was then deposited as an insulation layer, which itself was selectively opened (via reactive ion etching) at the electrode and connection sites. The devices were separated in another reactive ion etching step. The end product is a neural probe consisting of a shaft that is 380 µm wide, approx. 12 µm thick and 15 mm in length. The shaft contains 12 recording sites (15 × 15 µm²) and 4 stimulation sites (50 × 50 µm²). A large circular aperture, surrounded by a 300 nm thick platinum ring, was integrated at the tip of the shaft, which served as support structure for the insertion tool during implantation^62^. The connection site was designed to fit into conventional zero insertion force connectors.

### Surgical Procedure

All experiments were conducted following protocols approved by the responsible Animal Care Committee of the Regierungspräsidium Freiburg (Permit G13/51) in accordance with the NIH guide for the care and use of laboratory animals^63^. Sprague Dawley rats (∼300 g) (Charles River), housed in groups of 2, under standard lighting (12 h light-dark cycle), and at 22 °C room temperature and 40% humidity were used in this study. Access to food and water was allowed *ad libitum*.

Aseptic preparation techniques were used in all surgical procedures described. The rats were exposed to a burst dose of inhalation anesthetic (4% isoflurane and 1–2 l/min O_2_) to ensure ease of positioning into the stereotaxic frame, after which reflexes were tested and confirmed to be non-evokable by the pinch test. Animals were further anaesthetized with isoflurane (1-1.5% and 1.5 l/min O_2_) during the course of the implantation procedure by a tight fit nose cone. Once the rats were positioned in the stereotaxic frame (Model 900, Kopf Instruments, USA), skulls were disinfected and fur was shaved for ease of access to the trepanation site and to avoid healing complications from hair and dander. Eyes were covered with a moistening cream (Bepanthen, Bayer A.G., Germany) to prevent drying related damage during the surgical procedure.

A midline incision was made using a sterile scalpel. The subcutaneous tissue was removed using a blunt tip and the trepanation area was cleaned. Coordinates (from Bregma^64^) for the trepanation were AP: 0 and −3.0 mm, with ML: ±1.5 mm, and DV: −3 mm. Trepanations were performed using a handheld dental microdrill with a 0.9 mm drill bit. Drilling was done in short bursts and the progress was monitored carefully to prevent damage to the dura. To prevent over-heating, the surface of the skull was flooded with 0.9% NaCl solution (B. Braun, Germany), which was periodically refreshed to maintain the temperature. The pilot hole was then widened to facilitate ease of probe insertion. Once the trepanation was complete, the meninges were carefully removed using a 23G cannula (B. Braun, Germany) with a nicked tip. Care was taken to prevent damage to the cortical layers by this tip. All bleeding was stemmed and cleaned before the probes were implanted at the sites of interest.

Probe insertion was performed using a protocol adapted from Richter *et al*.^62^. The probe was ethanol disinfected, air dried, and laid over the implantation site, which was moistened using 0.9% NaCl (B. Braun, Germany). Once the probe was in position, a ceramic ferrule with a glass fiber tip (105 µm diameter; CFMLC21L02, Thorlabs, USA) was used for the insertion process.

Once flexible probes were implanted and secured in place on the skull, the skin was closed using surgical grade skin adhesion glue. Animals were allowed to recover for 1 h before placing them back into the animal facility. The animals were treated with Carpofen (5 mg/kg intraperitoneal; Carprieve, Bayer A.G., Germany) for 4 days post implantation for pain relief. Animals were monitored for infection or abnormal behavior over the whole experimental timeline. Subsequently, rats were transcardially perfused with 0.9% saline followed by perfusion with 600 ml fixative (4% paraformaldehyde in 0.1 M phosphate buffer, pH 7.4) for 12 min. After carefully removing the brain, tissue was immersion post-fixed in the same fixative overnight at 4 °C.

### Tissue processing

After washing with 0.1 M phosphate buffer, small cubes of tissue were dehydrated in a graded series of ethanol and embedded in LR-White (ScienceServices, Germany). X-ray imaging was performed at the BAMLine (BESSY II, Berlin, Germany) on an acute tissue sample (4 hpi) as well as a chronic tissue sample (12 wpi). In order to be able to evaluate the image quality with a sample that did not contain a probe, control tissue (rat cortex without an implanted probe) was also investigated.

A second set of samples (n = 3, all at 2 wpi) was imaged at the DESY imaging beamline (IBL, Hamburg, Germany) after contrasting of the tissue with Osmium (OsO_4_). For this set of samples, the tissue blocks were osmicated with 0.5% OsO_4_ in 0.1 M phosphate buffer for 30 min, stained with 1% uranyl acetate in 70% ethanol for 30 min, dehydrated in graded ethanol, and embedded in epoxy resin (Durcupan ACM, Sigma-Aldrich, USA). Sample blocks were trimmed to an edge length of approx. 2 mm along the xy-plane to keep the sample dimensions as close to the size of the field of view of µCT as possible in order to obtain optimal data quality. Samples were approx. 1 cm in height to be able to fix them on the sample holder for X-ray imaging.

After X-ray imaging embedded tissue blocks were sectioned parallel to the cortical surface with a diamond knife (Diatome; 500 nm section thickness), collected on glass slides, stained with 1% toluidine blue in 1% sodium borate and coverslipped using Polymount (Polysciences, Inc.). Bright field light micrographs were obtained using a Zeiss Axioplan 2 microscope.

### X-ray imaging

Tomographic imaging of the first set of samples was performed at the synchrotron X-Ray facility BAMLine (BESSY II, Berlin, Germany). A Si-W multilayer monochromator was used to produce a monochromatic X-ray beam with an energy of 19 keV and an energy resolution of ΔE/E = 10^−2^. A cadmium tungstate scintillator screen converted the X-rays into visible light. The optical lens system in combination with a CCD-camera (PCO 4000 camera, 4008 × 2672 pixels, PCO, Germany) covered a field of view of 1.8 × 1.2 mm^2^. The pixel size of the system was 438 nm in all dimensions. 2200 projections and 230 flatfields were measured over an angular range of 180° with an exposure time of 2.2 s. For edge enhanced imaging the samples were scanned in phase contrast mode with a specimen – detector distance of 75 mm. The tomographic raw data was corrected by the subtraction of the measured background of the CCD-camera and each radiogram was normalized with the highest correlating flatfield by using an IDL-based (interactive data language) program (Harris Geospatial Solutions, USA). The phase information of the propagation-based phase contrast was retrieved by application of a Fourier filter based on a single-distance phase-retrieval algorithm^65^. For the tomographic reconstruction an implementation of a filtered back projection algorithm, the IDL-based library called “gridrec”^66^, was used.

The second set of samples was scanned at the IBL (DESY, P05, Hamburg, Germany). The selected energy was 31 keV. The effective pixel size was 574.24 nm. For a comparable phase contrast the specimen-detector distance was increased to 202 mm. 1201 projections were taken over an angular range of 360° with an exposure time of 1.2 s, using an EHD SC09000M CCD camera (EHD imaging, Germany). For the tomographic reconstruction of the second set of samples the MATLAB-based (The MathWorks, USA) library “Astra Toolbox”^67^ was used. Finally, the reconstructed data was filtered with a non-local means denoising filter^68^.

### Image processing and segmentation

The µCT-datasets were processed as 16-bit TIFF image series. Data from the BAMLine (438 nm voxel size) was downscaled to a voxel size of 876 nm (isotropic) and reduced to 8-bit images in order to reduce the dataset size. Data from the IBL-beamline was similarly 2-2-2-binned (1148 nm isotropic voxel size) and converted to 8-bit images. No visible loss of contrast was observed by reducing the bit-depth. The datasets were rotated and cropped in order to obtain volumes with a centered probe and without expendable information outside of the tissue. The data of the first image set spanned 1223 × 886 × 817 µm³ (control tissue), 613 × 965 × 895 µm³ (12 wpi), and 1121 × 1388 × 701 µm³ (4 hpi). Of the second set two tomograms were analyzed quantitatively, which had a volume of 799 × 815 × 1743 µm³ (2 wpi sample a), and 1738 × 1743 × 831 µm³ (2 wpi sample b). In all grayscale images shown in this work brightness and contrast have been equally adjusted over the complete image in order to highlight the structures to be displayed.

Segmentation of the datasets was performed using ImageJ and MATLAB. By using appropriate image filters the tomographic data was altered in order to increase the contrast of the structure to be segmented. Via thresholding a segmentation of the structure of interest was then obtained. The tool CTvox (Bruker microCT, Belgium) was used for visualization of the 3D reconstruction of segmented data. Quantification of the datasets was performed by MATLAB as explained in the results section. Both raw and processed tomographic imaging data can be provided to readers upon request. Please note that due to the large data size (approx. 80 GB per unprocessed tomographic dataset), high bandwidth internet connection or sending data on a hard drive will be required.

### Raman spectroscopy

Raman spectroscopy was performed on embedded tissue samples after preparing smooth block face surfaces by trimming the specimen using an ultramicrotome (PowerTome, RMC Boeckeler, USA). The samples were analyzed using a WITec alpha 300 confocal Raman microscope (WITec, Germany) with a 532 nm laser operated at 5 mW. The laser beam was focused on the sample and collected by a water immersion objective (Zeiss W Plan-Apochromat 63x/1.0). A WITec UHTS VIS-NIR spectrometer with a 600 gr/mm optical grating and with a Peltier-cooled CCD-camera was used to measure Raman spectra at a spectral resolution of approx. 4 cm^−1^. Single spectra were integrated for 10 s and accumulated 5 times. Background subtraction was performed to remove fluorescence.

### SEM and EDX

SEM imaging and EDX mapping were performed on a TESCAN VEGA3 (TESCAN, Czech Republic). The sample was prepared for imaging equally as for Raman spectroscopy. In addition, a thin gold layer was deposited on the sample block face by sputtering to render the sample conductive. The microscope was operated at 15 kV with a secondary electron detector. EDX-mappings were acquired at the same acceleration voltage using a built-in EDAX TEAM Element (AMETEK, USA) EDX-system with a dwell time of 500 µs/pixel and 32 frame accumulations.

## Supplementary information

Supplementary material
