# Supplementary material for: Quantitative synchrotron X-ray tomography of the material-tissue interface in rat cortex implanted with neural probes
Source: Sci Rep. 2019 May 21;9:7646. doi: 10.1038/s41598-019-42544-9 (PMC6529414; doi:10.1038/s41598-019-42544-9)
Supplement: Supplementary file 1 — Supplementary material [file 41598_2019_42544_MOESM1_ESM.docx]

# Supplementary information to:

# Quantitative synchrotron X-ray tomography of the material-tissue interface in rat cortex implanted with neural probes

Thomas Böhm^1,2,3^, Kevin Joseph^3,4^, Matthias Kirsch^3,5^, Riko Moroni^1,2^, André Hilger^6^, Markus Osenberg^6,7^, Ingo Manke^6^, Midori Johnston^3,8^, Thomas Stieglitz^3,9,10^, Ulrich G. Hofmann^3,4^, Carola A. Haas^3,8,10^, and Simon Thiele^1,2,3,11,12,^*

1 Laboratory for MEMS Applications, IMTEK Department of Microsystems Engineering, University of Freiburg, Georges-Köhler-Allee 103, 79110 Freiburg, Germany

2 Freiburg Center for Interactive Materials and Bioinspired Technologies (FIT), University of Freiburg, Georges-Köhler-Allee 105, 79110 Freiburg, Germany

3 BrainLinks-BrainTools, University of Freiburg, Georges-Köhler-Allee 80, 79110 Freiburg, Germany

4 Neuroelectronic Systems, Dept. of Neurosurgery, Faculty of Medicine, University Medical Center, Engesserstraße 4, 79108 Freiburg, Germany

5 Department of Neuroanatomy, Institute of Anatomy and Cell Biology, Faculty of Medicine, University of Freiburg, Albertstraße 23, 79104 Freiburg, Germany

6 Helmholtz Center Berlin for Materials and Energy, Hahn-Meitner-Platz 1, 14109 Berlin, Germany

7 Institute of Materials Science and Technology, Technical University Berlin, Hardenbergstraße 36, 10623 Berlin, Germany

8 Experimental Epilepsy Research, Dept. of Neurosurgery, University Medical Center, Breisacher Straße 64, 79106 Freiburg, Germany

9 Laboratory for Biomedical Microtechnology, IMTEK Department of Microsystems Engineering, University of Freiburg, Georges-Köhler-Allee 102, 79110 Freiburg, Germany

10 Bernstein Center Freiburg, Hansastraße 9a, 79104 Freiburg, Germany

11 Forschungszentrum Jülich GmbH, Helmholtz-Institute Erlangen-Nürnberg for Renewable Energy (IEK-11), Egerlandstraße 3, 91058 Erlangen, Germany

12 Department of Chemical and Biological Engineering, Friedrich-Alexander-Universität Erlangen-Nürnberg, Egerlandstraße 3, 91058 Erlangen, Germany

*Corresponding author

**Corresponding author:** Simon Thiele; Egerlandstraße 3, 91058 Erlangen, Germany; +49 9131 85-20843; si.thiele@fz-juelich.de


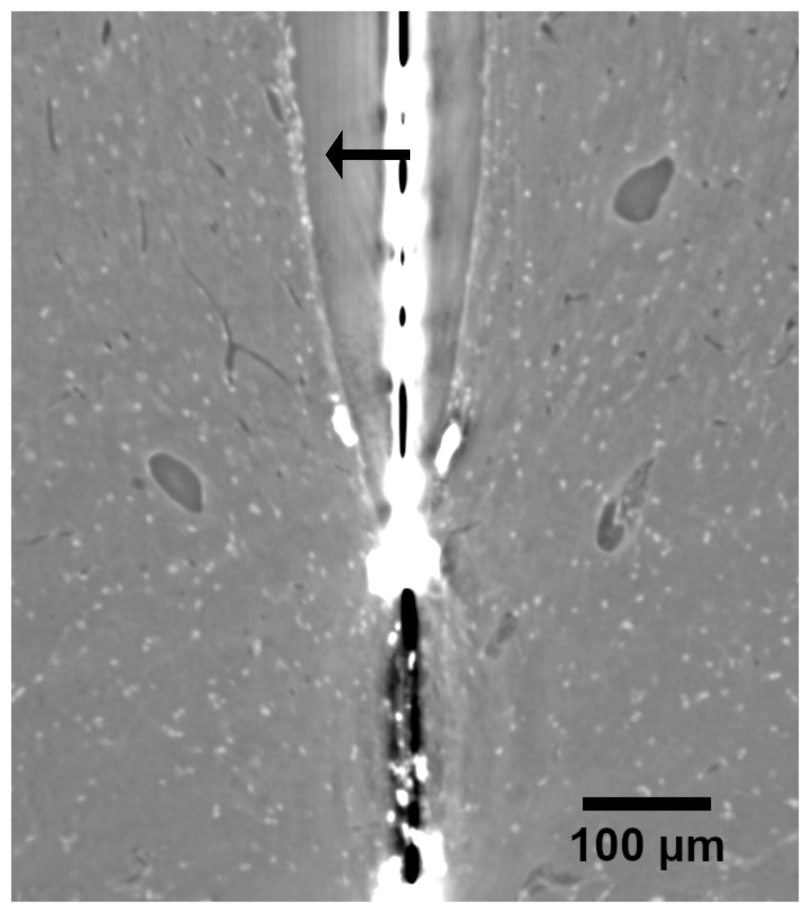


Supplementary Fig. 1: Virtual vertical section of rat cortex tissue 12 wpi. Adjacent to the elliptically shaped empty area around the probe a higher density of cells can be seen (black arrow).


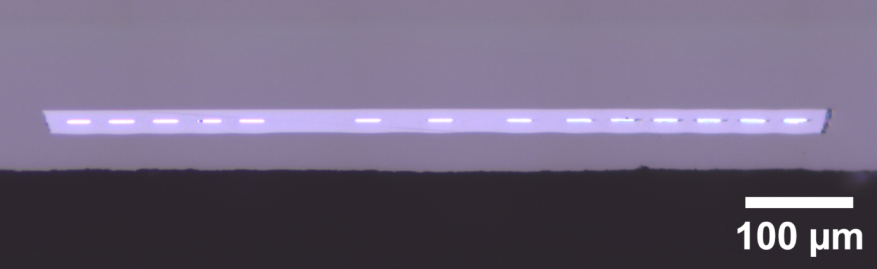


Supplementary Fig. 2: Light microscopic image of a probe embedded in pure epoxy resin (Durcupan). The cross-section of the sample was prepared with an ultramicrotome. No separation between probe and polymer is visible around the probe.


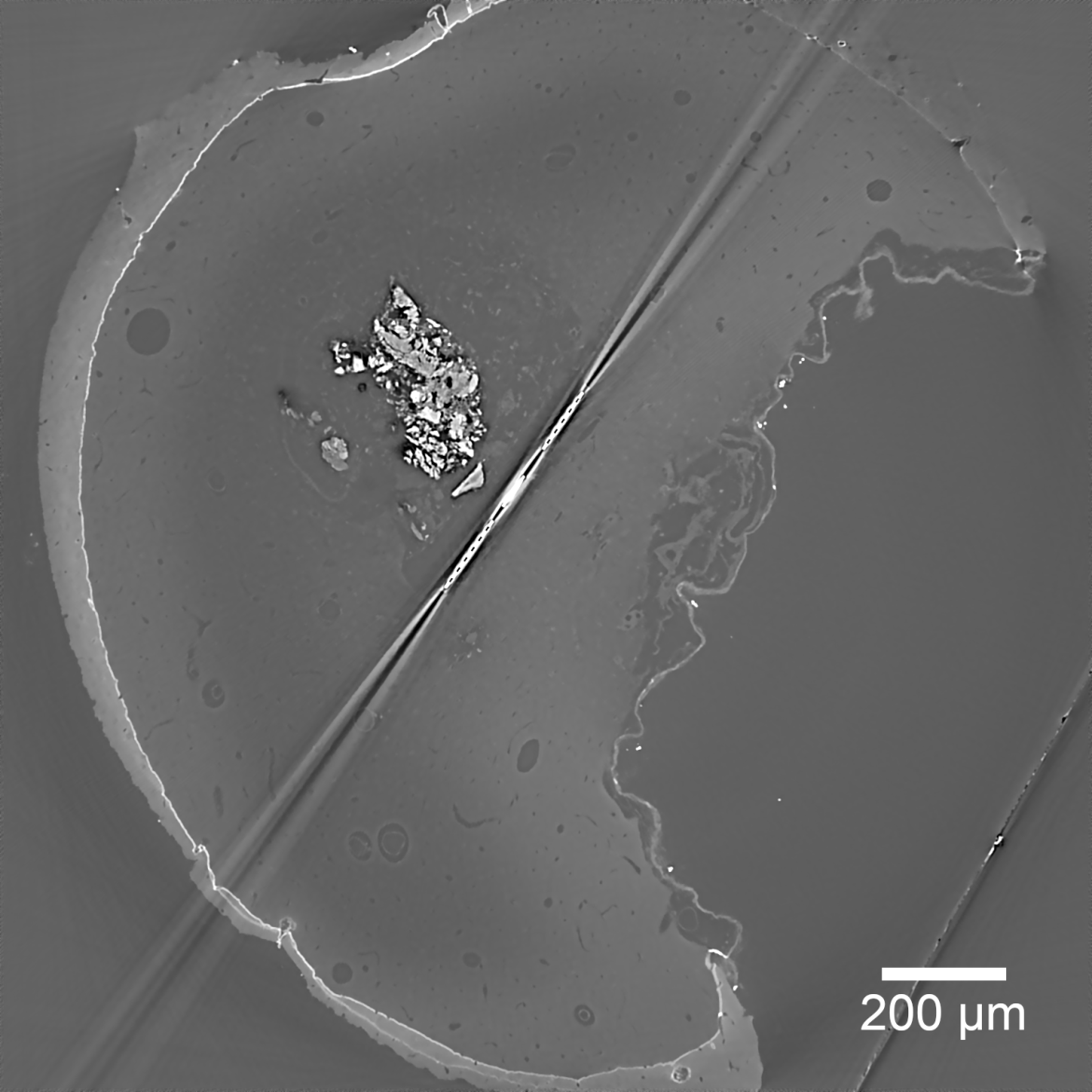


Supplementary Fig. 3: Virtual transversal section of rat cortex tissue of a tomogram from the second data set (2 wpi sample a). The tissue was contrasted with OsO_4_, which did not stain the sample homogeneously, leading to a high contrast at the edges but a low contrast in the sample center. The SNR in this tomogram was insufficient for a reliable segmentation and analysis of cells and blood vessels. Nevertheless, the contrast between probe and calcification allowed a clear distinction between these structures and the surrounding tissue.


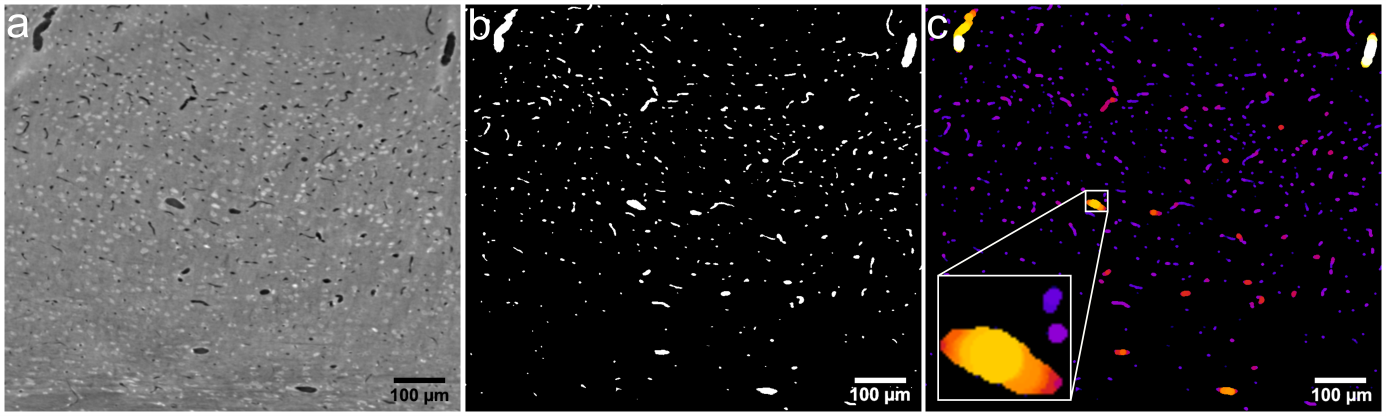


Supplementary Fig. 4: Scheme of the workflow for size distribution calculations of blood vessels. The grayscale X-ray dataset (a) was segmented (b) and afterwards the diameter of the vasculature was determined by projecting spheres into the segmented space, each with the largest diameter that is possible without penetrating into the surrounding volume. In c) a simplified 2D representation is provided, with circles instead of spheres and the diameter of the circles represented by the pixel color (larger diameters are represented in yellow; smaller diameters in blue). The inset in c) is a 6x magnified view on the indicated image area.


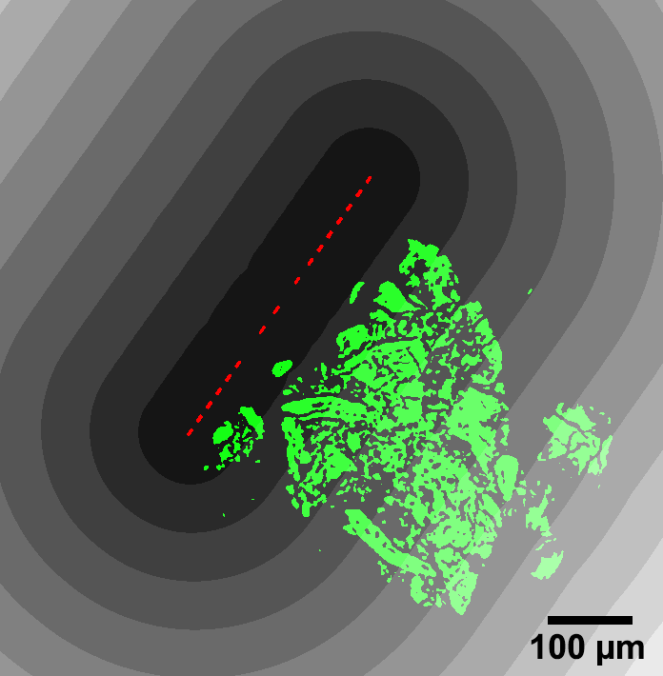


Supplementary Fig. 5: Scheme for distance calculations between different structures in a tomogram. Red: segmentation of neural probe; green: segmentation of calcification. The gray layers indicate distance intervals from the probe. Using these intervals, a distance distribution of the calcification with respect to the neural probe was obtained.
